# Trends in NIH-supported career development funding: implications for institutions, trainees, and the future research workforce

**DOI:** 10.1172/jci.insight.142817

**Published:** 2020-09-03

**Authors:** Marisa L. Conte, Santiago Schnell, Adrienne S. Ettinger, M. Bishr Omary

**Affiliations:** 1 Taubman Health Sciences Library and; 2 Department of Molecular & Integrative Physiology, University of Michigan Medical School, Ann Arbor, Michigan, USA.; 3 Office of Academic Affairs and Research, Rutgers Biomedical and Health Sciences, Rutgers University, Newark, New Jersey, USA.

A variety of circumstances over the last two decades have imperiled the capacity and future potential of the US biomedical research enterprise. A perspective from Bruce Alberts, Marc Kirschner, Shirley Tilghman, and Harold Varmus noted that “precipitous actions could damage a system that has served many scientists and the public well over the past several decades. However, it is equally dangerous to ignore the structural flaws that are generally acknowledged to have produced the current system” (1). An important aspect of securing future capacity is the need to recruit and retain a diverse group of trainees as the foundation and future for our biomedical research workforce and to provide training opportunities that equip trainees for productive and rewarding careers (2). The NIH, the major biomedical research funding agency in the United States, provides remarkable support for fellowships, training, and career development awards, in addition to supporting fundamental, translational, and clinical research with a total budget of $41.6 billion in fiscal year 2020 (5.9% increase from $39.3 billion during the 2019 fiscal year) (3).

We compared the ranking of institutions with the largest amount of NIH support for research at 2-year intervals during the even years 2010–2018 with the top-ranked institutions in NIH support for training and career development. This effort was based on the hypothesis that the two types of support may not necessarily go hand in hand, likely due to differing institutional efforts or success in efforts that promote supporting training (T type) grants and encourage trainees to seek their own independent training and career development (F and K type) support. We also summarize the 2010–2018 annual NIH funding for training as a percentage of total NIH funding, per the final appropriations. Our analysis showed that although the top 14 ranked institutions in research funding include the same top 14 top ranked in F/K/T support, there was identical ranking in only 5 of the 14 (36%) institutions, with the remaining 9 being either higher or lower. We discuss the implication of these findings and propose specific approaches by NIH and training institutions to further enhance training and career development opportunities for the next generation of our biomedical workforce.

## Why support training and career development?

The return on investment of career development support has been well documented. Several analyses examined the impact of National Research Service Award postdoctoral fellowships on subsequent NIH R01 outcomes for applicants who obtained fellowships between 1996 and 2008 (4–6). For example, Heggeness et al. found that NIH F32 postdoctoral fellowships increased the likelihood of receiving subsequent NIH-funded research awards in general and specifically of receiving subsequent R01 awards (4). Nikaj and Lund examined the trajectory of researchers who had been awarded K awards during the doubling period of NIH funding (years 1998–2003) and beyond (years 2004–2016) and found that K awardees were 24.1% more likely to obtain an R01, R01 equivalent, or Research Project Grant than researchers without these awards (5). Similarly, Pickett’s analysis showed that the percentage of investigators obtaining a first-time R01 who previously were awarded F or K awards has steadily increased since 2000 (6).

Within the different K categories, there are differences in future success. For example, K99 awardees are more successful in converting their K99 to an R01 within 5 years (30% for the analyzed K-awarding years of 2008–2012) or 7 years (48% for 2008–2010) compared with K01, K08, or K23 awardees (19%–23% and 30%–37%, for the years 2008–2012 and 2008–2010, respectively) (7). These differences indicate that some career development mechanisms appear to be more predictive of future success than others when using subsequent R01 funding as a performance indicator. Notably, there are substantial differences (and potential disparities) in the geographic distribution of K awards (7) (and R conversions, which Pickett addresses in greater depth in ref. 6). Furthermore, women and racial and ethnic minorities represent a higher percentage of new and early-stage investigators as compared with experienced investigators. These disparities suggest a need not only to increase such career awards but also to continue to provide mentoring and continued milestone-based support (8).

## Funding analysis

We examined funding rates for training and career development by focusing on the NIH F, K, and T mechanisms within a fiscal year (collectively referred to as “training”). We note that some R-type grant mechanisms also provide training, but they were not included in the analysis. The F/K/T mechanisms are broadly understood as training and career development grants, but each has a specific focus (9): F grants are fellowship programs and include specific funding for international, predoctoral, postdoctoral, and MD/PhD researchers; for special topics (e.g., medical informatics); for more established researchers looking to change career fields; or for intramural researchers at the NIH or FDA. K grants focus on career development for research scientists and clinician-scientists in early or midcareer. T awards support research training programs (e.g., discipline specific or in service to various groups).

Total research and other NIH grant dollars awarded to academic US medical centers were obtained from the Blue Ridge Institute for Medical Research (BRIMR) annual data (10). NIH training funding data were downloaded from the NIH RePORT Expenditures and Results Tool: https://projectreporter.nih.gov/reporter.cfm The search criteria retrieved all F/K/T awards by fiscal year for the even years 2010–2018 from all NIH Institutes and Centers. The collected data did not include American Recovery and Reinvestment Act–funded projects, and projects with suffixes S or X were manually excluded. Institution names were standardized to match the NIH institution, with the exception of Vanderbilt University (years 2016, 2018). For this study, only institutions that are also represented in the BRIMR rankings are included.

## Comparison of total research dollars versus training dollars

**Figure 1:**
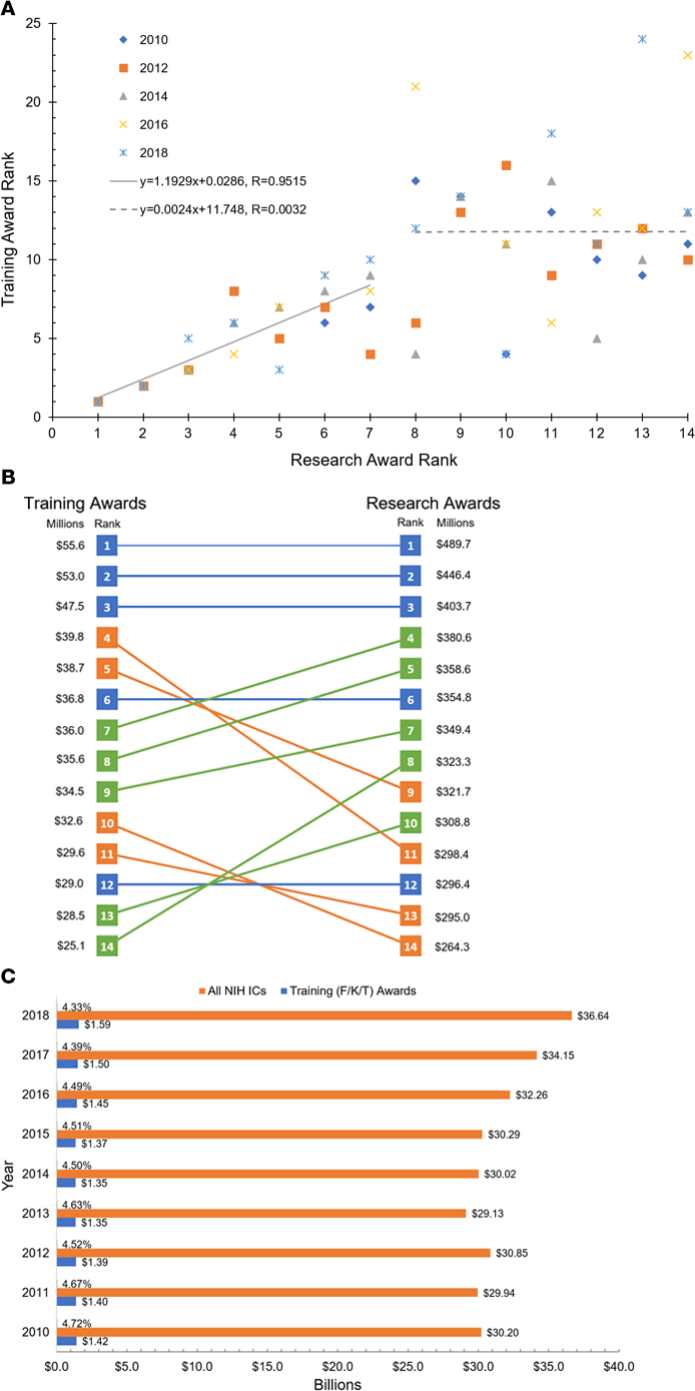
NIH research and training support to top 14 awarded institutions and in total during 2010–2018. (**A**) Relationship between training and research awards for the 14 highest average training (F/K/T) and research awards (years 2010–2018). There is a positive linear relationship between the training and research award ranks for the top 7 institutions. The data follow a trend line with *R* = 0.9515. This relationship vanishes from the eighth ranked institution onward, and no apparent correlation is found for the training and research awards. (**B**) Paired institutional rankings for the 14 highest average training (F/K/T) and research awards in millions of dollars (years 2010–2018); blue indicates same rank for training and research awards, orange indicates higher rank for training awards, and green indicates higher rank for research awards. (**C**) Total training (F/K/T) awards as a percentage of all NIH Institutes and Centers (ICs) in billions of dollars (years 2010–2018).

The top 14 schools by BRIMR ranking comprise 39.3% of total awarded NIH dollars to BRIMR-listed institutions and 36.5% of total training dollars through F/K/T awards. Of the top 14 schools, training-type grants were between 7.7% and 13.3% of their total NIH awards for the period of analysis (see Supplemental Table 1; supplemental material available online with this article; https://doi.org/10.1172/jci.insight.142817DS1). In general, when averaged over the entire 2010–2018 period (see Supplemental Table 1 for individual and averaged years), there was a strong correlation between training and total BRIMR research support for the top 7 schools that no longer persisted for the schools ranked 8–14 (Figure 1A). There was a 1:1 match between the BRIMR rank and average training support rank for the first 3 of the top 14 schools, and 2 additional schools also held the same rank for both NIH total and training ranks (Figure 1B). Four schools ranked higher for their average NIH training support than for their rank of overall NIH awards, and 5 ranked higher for overall NIH awards (Figure 1B). We focused on the top-funded 14 institutions because they were within the top 14 in both training and total NIH funding, but as noted, even within this group there was a mismatch. This mismatch also carries over beyond the top 14 schools (Supplemental Table 1 and data not shown).

We also retrieved the 2010–2018 annual NIH funding for training and career development and for total NIH funding (3, 11). Of note, the percentage support for training and career development, as compared with total NIH budget, has been gradually decreasing since 2010 (Figure 1C). For example, training support in 2010 was 4.72% of the total NIH budget but decreased to 4.33% in 2018. The total dollars for all NIH ICs have increased on average 22% between the period 2010–2015 and 2018, with annual increases since 2016, while the total NIH training and career development awards remained on average flat, around $1.42 billion. In terms of training support, there is a huge multiplier effect for small percentage changes; for example, a difference of only 0.01% in the 2018 total NIH budget corresponds to $3.7 million, which can support 54 postdoctoral fellowships (using the 2020 NIH annual pay scale averaged for years 1–3, a benefit rate of 20%, and an indirect rate of 8%). Thus, the small decrease in the percentage of the total budget invested in training may have a marked impact on the pipeline of future investigators.

Our analysis has several limitations. In the BRIMR data for the total NIH rankings, we are excluding from the analysis institutions that are not academic medical centers per se, although they may be affiliated or partner with an academic medical center. Additionally, institutions may have several Data Universal Number System numbers that are used for grant registration and, thus, are not receiving “credit” for grants awarded under one umbrella organization. For these reasons, BRIMR rankings are sometimes contested by the affected institutions. However, we believe that the comparison for the BRIMR top 14 institutions we used provides relevant comparisons within their institutions. Additionally, the K award mechanism (e.g., K24) is not limited to early career investigators, and some R grant mechanisms that have training components (e.g., R25, R36, R38, R90) were not included in our analysis because the bulk of the training and career development awards are encompassed by the F/K/T mechanisms. Finally, in looking only at total dollars awarded, we acknowledge that this is a limited picture of training activity at any institution.

## Where do we go from here?

The analysis herein suggests future actions to be considered by training institutions and the NIH. *First*, we focused on the top 14 BRIMR-ranked institutions, though the listing has more than 140 entries (albeit in some cases more than one entry might be within the same institution). One limitation of our analysis is that it does not include the numbers of potential mentors and trainees, which are not readily available. Regardless, the findings may serve to alert institutions that have a high rank gap in overall NIH funding compared with training support to consider committing more resources to early career investigators and trainees. *Second*, there is an opportunity for institutions and the NIH to arrange national-scale meetings to share best practices and devise new programs. One NIH R13-supported meeting resulted in the 2017 report on the future of graduate and postdoctoral training in the biosciences (12). Such meetings are needed to chart the course beyond this decade and to converge excitement and intention with necessary action. *Third*, our analysis has implications not only for institutions but also for trainees and those aspiring for a biomedical research career. For example, success rates for F and K award trainees are predictive of future first R01 awards, and institutions generally have a strong preference for hiring or retaining K awardees (4–7). Hence, strong training programs have value both to trainees and to their prospective hiring institutions, and early career researchers may wish to identify institutions that are more likely than others to help propel them into research careers. *Fourth*, institutions can increase their long-term footprint and impact in the biomedical research enterprise by establishing training programs that help early career biomedical scientists to successfully secure NIH F or K or other nonfederal career awards. *Fifth*, the NIH can further enhance the critical role it has been playing by allocating additional funds to support training and career development nationally, based on percentage of total research funding, particularly given the concern that the welcome increase in total NIH budget has not been matched proportionately by support for new or expanded training-type funding mechanisms. The NIH can also play a key role in analyzing the diversity of where and to whom training and career development support is directed, which can provide fresh ideas on how to diversify the biomedical research workforce.

Finally, given that the COVID-19 pandemic has halted the training of NIH-supported F/K/T awardees, particularly those undertaking wet lab or direct non–COVID-19 patient contact research, it is essential to provide additional stimulus funds to help make up for what is likely to be at least several months of lost time (13). With the tremendous growth in the biomedical research enterprise during the past three decades (e.g., the NIH budget in 1990 was $7.6 billion; ref. 3), the historically highly successful approach and support by the NIH for career development is now at a stage that warrants further “development.”

## Supplementary Material

Supplemental Table 1

## Version 1 09/03/2020

Electronic publication
